# Whole-body magnetic resonance imaging (WB-MRI) reporting with the METastasis Reporting and Data System for Prostate Cancer (MET-RADS-P): inter-observer agreement between readers of different expertise levels

**DOI:** 10.1186/s40644-020-00350-x

**Published:** 2020-10-27

**Authors:** Paola Pricolo, Eleonora Ancona, Paul Summers, Jorge Abreu-Gomez, Sarah Alessi, Barbara Alicja Jereczek-Fossa, Ottavio De Cobelli, Franco Nolè, Giuseppe Renne, Massimo Bellomi, Anwar Roshanali Padhani, Giuseppe Petralia

**Affiliations:** 1 Division of Radiology, IEO European Institute of Oncology IRCCS, via Ripamonti 435, 20141 Milan, Italy; 2 Postgraduate School of Diagnostic and Interventional Radiology, University of Milan, 20122 Milan, Italy; 3 Department of Medical Imaging, Sunnybrook Health Science Centre, Toronto, Canada; 4 Division of Radiotherapy, IEO European Institute of Oncology IRCCS, via Ripamonti 435, 20141 Milan, Italy; 5 Department of Oncology and Hemato-Oncology, University of Milan, 20122 Milan, Italy; 6 Division of Urology, IEO European Institute of Oncology IRCCS, via Ripamonti 435, 20141 Milan, Italy; 7 Division of Urogenital and Head and Neck Tumours Medical Treatment, IEO European Institute of Oncology IRCCS, via Ripamonti 435, 20141 Milan, Italy; 8 Division of Uropathology and Intraoperative Diagnostics, IEO European Institute of Oncology IRCCS, via Ripamonti 435, 20141 Milan, Italy; 9 Paul Strickland Scanner Centre, Mount Vernon Cancer Centre, Northwood, England; 10 Precision Imaging and Research Unit - Department of Medical Imaging and Radiation Sciences, IEO European Institute of Oncology IRCCS, via Ripamonti 435, 20141 Milan, Italy

**Keywords:** Whole body MRI, Prostate cancer, Inter-observer agreement, MET-RADS-P

## Abstract

**Background:**

The METastasis Reporting and Data System for Prostate Cancer (MET-RADS-P) guidelines are designed to enable reproducible assessment in detecting and quantifying metastatic disease response using whole-body magnetic resonance imaging (WB-MRI) in patients with advanced prostate cancer (APC).

The purpose of our study was to evaluate the inter-observer agreement of WB-MRI examination reports produced by readers of different expertise when using the MET-RADS-P guidelines.

**Methods:**

Fifty consecutive paired WB-MRI examinations, performed from December 2016 to February 2018 on 31 patients, were retrospectively examined to compare reports by a Senior Radiologist (9 years of experience in WB-MRI) and Resident Radiologist (after a 6-months training) using MET-RADS-P guidelines, for detection and for primary/dominant and secondary response assessment categories (RAC) scores assigned to metastatic disease in 14 body regions. Inter-observer agreement regarding RAC score was evaluated for each region by using weighted-Cohen’s Kappa statistics (K).

**Results:**

The number of metastatic regions reported by the Senior Radiologist (249) and Resident Radiologist (251) was comparable. For the primary/dominant RAC pattern, the agreement between readers was excellent for the metastatic findings in cervical, dorsal, and lumbosacral spine, pelvis, limbs, lungs and other sites (K:0.81–1.0), substantial for thorax, retroperitoneal nodes, other nodes and liver (K:0.61–0.80), moderate for pelvic nodes (K:0.56), fair for primary soft tissue and not assessable for skull due to the absence of findings. For the secondary RAC pattern, agreement between readers was excellent for the metastatic findings in cervical spine (K:0.93) and retroperitoneal nodes (K:0.89), substantial for those in dorsal spine, pelvis, thorax, limbs and pelvic nodes (K:0.61–0.80), and moderate for lumbosacral spine (K:0.44).

**Conclusions:**

We found inter-observer agreement between two readers of different expertise levels to be excellent in bone, but mixed in other body regions. Considering the importance of bone metastases in patients with APC, our results favor the use of MET-RADS-P in response to the growing clinical need for monitoring of metastasis in these patients.

## Background

Advanced Prostate Cancer (APC) typically demonstrates a good initial response to androgen deprivation therapy (ADT), but in the course of 1 to 3 years, almost inevitably progresses into metastatic Castration Resistant Prostate Cancer (mCRPC) [1–3]. Evolution of mCRPC tumors may lead to an Androgen Receptor (AR) independent phenotype [4] and loss of prostate cancer markers such as prostate-specific antigen (PSA) expression. In addition, lineage plasticity and, in some cases, the expression of small cell/neuroendocrine features [4, 5], may result from the multiclonal and heterogeneous tumor proliferation [6]. Although the hypothesis that each metastasis originates from a single tumor cell is generally supported, a recent study has demonstrated evidence for the existence of polyclonal seeding in the context of androgen-deprived metastatic prostate cancer [7]. Due to differences in the susceptibilities of the subclones, therapy has the effect of selecting for resistant tumor subclones, and this leads to heterogeneous metastatic disease. New therapies developed for the management of mCRPC, have improved [8, 9] survival in this patient population.

The early identification of treatment failure in men with mCRPC on systemic therapy could spare them unnecessary treatment and potential toxicity while reducing the costs of ineffective treatments and decreasing the time to initiation of a potentially effective, next-line treatment [5]. With up to 30% of cancers developing radiographic progression without progression in PSA or clinical symptoms [10], there is considerable interest in identifying imaging techniques able to effectively monitor systemic disease in APC patients. In light of the evolving status of tumor sub-clones in different anatomical sites in APC, the process of monitoring must also capture heterogeneity in metastases appearances and responses.

Next generation imaging techniques have better accuracy for detecting metastases than computed tomography (CT) and bone scintigraphy (BS) [5]. Moreover, as whole body imaging techniques that are able to capture disease heterogeneity, whole-body magnetic resonance imaging (WB-MRI) and positron emission tomography (PET) also hold the promise of being more accurate for evaluating treatment responses of bone disease [5]. The use of WB-MRI in oncology has grown under recommendations in several international guidelines [11]. In particular, it has been reported that WB-MRI can detect bone metastases with higher sensitivity than BS and with at least comparable performance to choline PET/CT [9]. Importantly, WB-MRI provides a clearer differentiation of bone metastases response, whereas bone scans and sodium fluoride PET/CT scans can only identify disease progression [12]. However, in the literature, the approaches to WB-MRI very widely and its use has largely been confined mainly to expert centers, causing some concerns about its broader applicability [13].

**Fig. 1:**
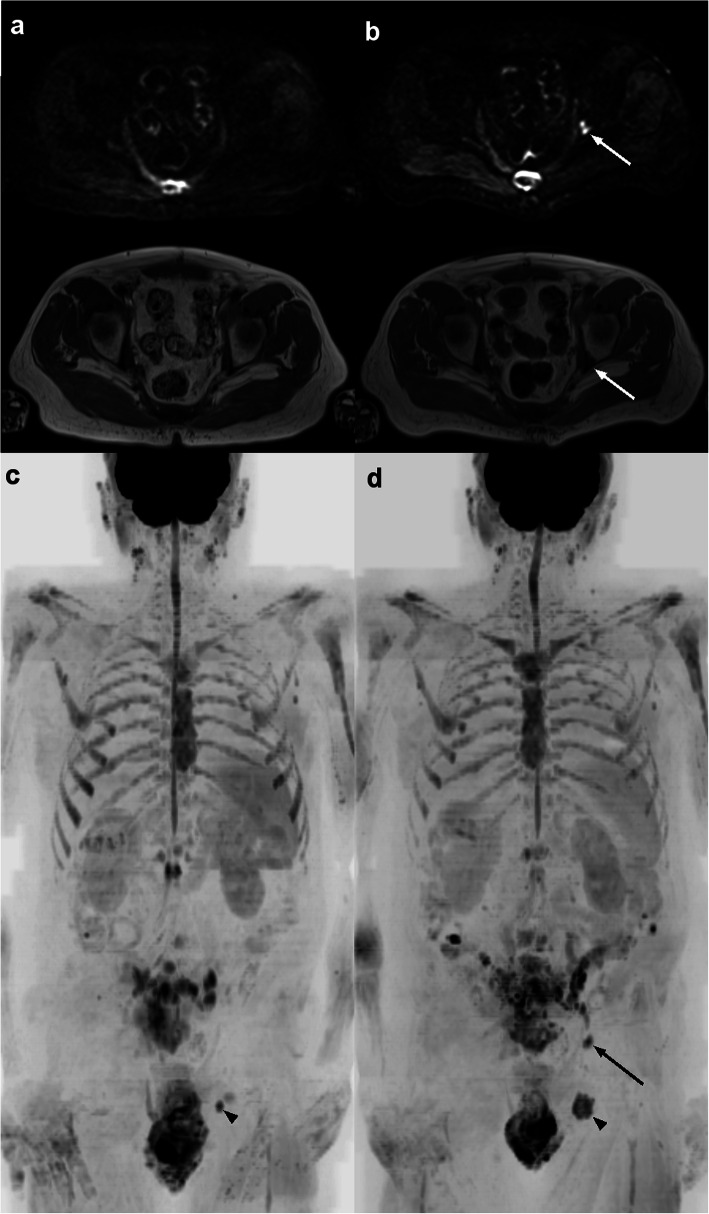
Example images of a 69-year-old man with castration resistant prostate cancer in progression, with primary/dominant RAC 5. **a**) and **b**) In the course of abiraterone treatment, axial diffusion-weighted (DW) b900 images (upper) and T1-weighted (T1) images (lower) show the appearance of a small acetabular lesion on the left. Inverted grayscale maximum intensity projection of the **c**) pre- and **d**) post-treatment b900 images, respectively illustrate the appearance of the left acetabular lesion (arrow) and an increase in size of an existing pelvic bone lesion (arrow head)

**Fig. 2:**
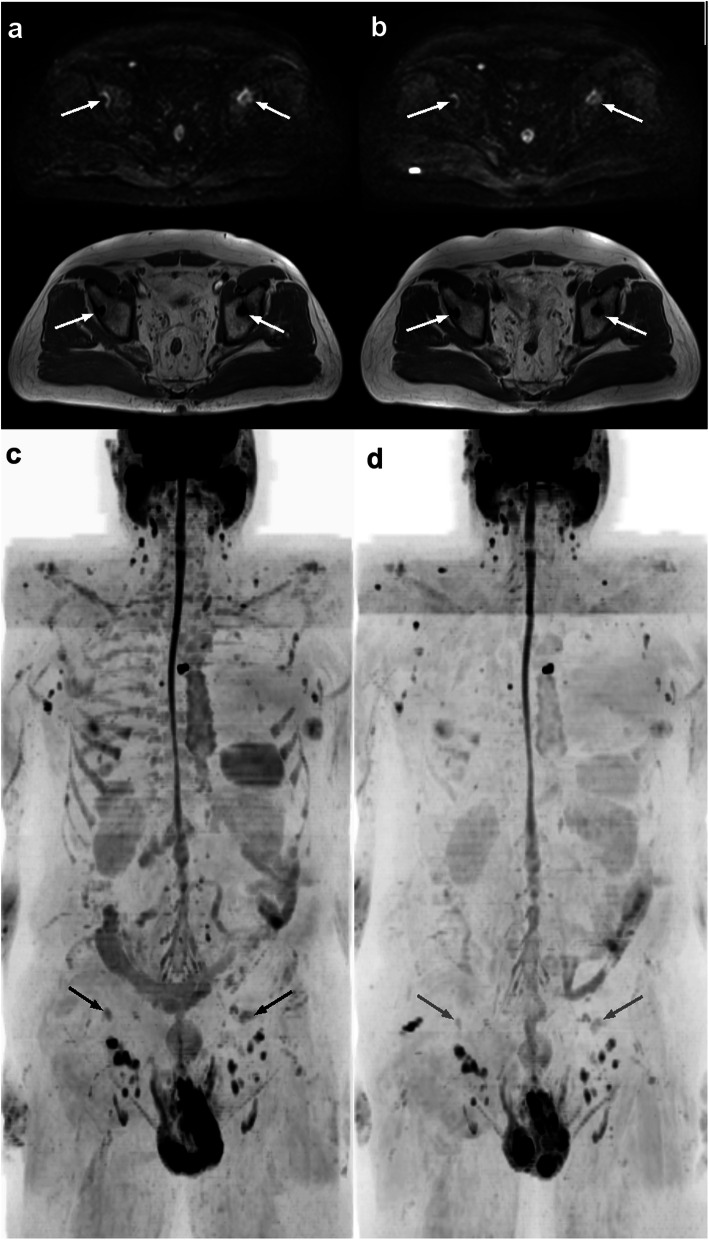
Example images of a 76-year-old man with advanced prostate cancer that is not progressing, with primary/dominant RAC 3. **a**) Axial DW b900 images (upper) and T1 images (lower) show the presence of lesions in pelvic bone (arrows) that are unchanged in **b**) follow-up MRI. The **c**) initial and **d**) follow-up b900 maximum intensity projection images confirm stability of disease throughout the body

**Fig. 3:**
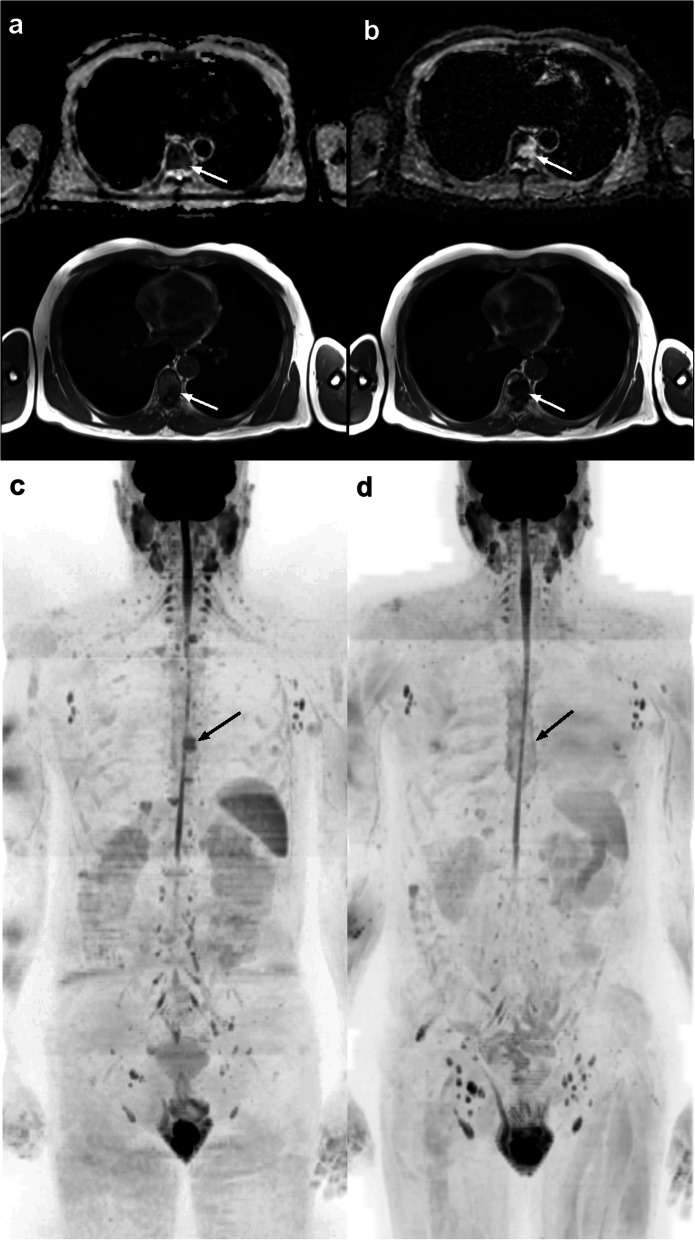
Example images of a 67-year-old man with metastatic hormone sensitive prostate cancer showing response, with primary/dominant RAC 1. **a**) Axial ADC map (upper) and T1 images (lower) at the start of luteinizing hormone releasing hormone agonist therapy show presence of a dorsal (T8 level) spine lesion with ADC value = 784 μm^2^/sec. **b**) Despite the follow-up T1 image (lower image) showing an increase in the lesion size due to the presence of edema accompanied by an increase in the ADC value = 1608 μm^2^/sec of the T8 lesion (arrows), suggestive of highly likely response. Three-dimensional b900 maximum intensity projection images **c**) at start of therapy and **d**) at follow-up illustrate the disappearance of the T8 lesion

The recently published “METastasis Reporting and Data System for Prostate Cancer” (MET-RADS-P) guidelines [9] provide a structured basis for assessing disease extent and following-up APC patients on treatment. MET-RADS-P proposes the first guidelines for stratifying treatment response of metastatic bone disease that go beyond the limited clinical classification of progression and no progression currently employed [14], while also enabling documentation of heterogeneity of responses (mixed/discordant responses) at the regional level using RACs (Response Assessment Categories). For each anatomic region, the primary/dominant RAC pattern is the response (on the RAC 1–5 scale; with a score of 5 being progressive disease (Fig. 1), a score of 3 being stable disease (Fig. 2) and a score of 1 being response to treatment (Fig. 3), and scores of 4 and 2 representing likely progression and response respectively) seen in the majority of lesions within the region. The secondary RAC pattern records the second most frequent RAC response within the region [9]. To our knowledge however, there are no data as yet regarding its performance in clinical practice or the level of expertise needed when adopting the guideline. The purpose of this study was, therefore, to evaluate the inter-observer agreement of WB-MRI examination reports produced by readers of different expertise when using the MET-RADS-P guidelines.

## Methods

### Patients and data

We analyzed therapy response in 50 pairs of WB-MRI examinations performed on 31 APC patients with hormone sensitive and castration resistant disease who had underwent at least two WB-MRI examinations in our institution between December 2016 and February 2018 for the follow-up of anti-cancer therapy. Sixteen of the patients had one prior WB-MRI examination (32 scans total; 16 examination pairs), 11 patients had two prior examinations (33 scans total; 22 examination pairs), and four patients had three prior examinations (16 scans total, 12 examination pairs). Inclusion criteria were the presence of metastatic cancer in previous WB-MRI and ongoing follow-up with WB-MRI performed in our institution. The median age of the included patients was 68 years (range, 48–83 years). The local ethical committee approved this retrospective study, and waived the requirement for specific consent from the patients.

### Imaging technique

All WB-MRI examinations were performed on a 1.5 T MR scanner (Magnetom Avanto^fit^, Siemens Healthineers, Erlangen, Germany) equipped with works-in-progress software for slice-specific shimming (iSHIM, Siemens Healthineers, Erlangen, Germany). The WB-MRI acquisition protocol and post-processing of images (Supplementary Table E1) were compliant to MET-RADS-P guidelines. A typical cumulative WB-MRI data acquisition time per examination was 40 min.

### Image analysis

A Senior Radiologist with 9 years of experience in WB-MRI reported the findings using the MET-RADS-P guidelines. A Resident Radiologist, after 6 months of training in oncological WB-MRI, subsequently reported the WB-MRI examinations, blinded to the Senior Radiologist’s reports. The radiologists had access to the reports of prior examinations and the patients’ clinical and biochemistry data (bone pain, PSA, etc.). Where patients had more than one examination pair in the study, the radiologists were not blinded to their results regarding prior examination pairs. According to MET-RADS-P guidelines, the presence or absence of metastasis was reported for each of 14 body regions: primary disease, 7 skeletal regions, 3 nodal and 2 visceral sites and other sites [9]. For each anatomic site of metastasis, in the follow-up examination the primary/dominant and secondary RAC pattern were scored using a scale of 1–5 indicating the likely response category: 1 highly likely to be responding, 2 likely to be responding, 3 stable, 4 likely to be progressing, and 5 highly likely to be progressing using the criteria for response assessment of bone and soft tissue lesions provided in the MET-RADS-P guidelines [9]. Finally, we did a per-patient ad hoc analyses, evaluating the number of patients in which WB-MRI lead to a change in treatment and the agreement between two radiologists.

### Statistical analysis

MET-RADS-P based findings were summarized by site and the inter-observer agreement between the Senior Radiologist and Resident Radiologist for region-based disease detection, assignment of RAC scores to metastases, and patient management was evaluated for each region by weighted-Cohen’s Kappa statistics (K). Inter-observer agreement was interpreted as none to slight (K:0.01–0.20), fair (K:0.21–0.40), moderate (K:0.41–0.60), substantial (K:0.61–0.80) or excellent (K:0.81–1.00). Statistical analysis was carried out with SAS software, version 9.4. A *p*-value of < 0.05 was considered to be statistically significant.

## Results

### Patient demographics

The majority of patients had undergone radical prostatectomy (19 of 31 patients). Amongst these patients, a Gleason Score (GS) of 4 + 4 was the most common diagnosis (26%, *n* = 8 patients), followed by GS 4 + 3 (19%, *n* = 6 patients) and GS 4 + 5 (16%, *n* = 5 patients). The remaining 12 patients had been treated with radiotherapy. Further details of therapies in use at the time of the WB-MRI examination(s) are given in Table 1.

**Table 1:** Breakdown of Treatments by Systemic Therapies and Site of Radiotherapy

Metastatic Status	Therapy		# ExaminationPairs		Sites of Radiotherapy
			No RT^f^	With RT^f^	
mHSPC^a^ (*N = *27)	LHRH agonists		12	4	pelvic bone cervical/dorsal spine other nodes other sites
	TAB^c^ (antiandrogens + LHRH agonists)		6	1	prostate and pelvic bone
	TAB + CHT^d^		4		
mCRPC^b^ (N = 23)	Abiraterone or Enzalutamide		2	2	pelvic bone cervical/dorsal/lumbosacral spine
	Abiraterone or Enzalutamide + LHRH agonists		7	2	dorsal spine+ limbs pelvic bone, retroperitoneal + other nodes
	Abiraterone or Enzalutamide + LHRH agonists + CHT		2		
	CHT		4		
	Other	Radiometabolic (Radium 223)	1		
		Enzalutamide vs Placebo + ADT^e^	2		
		LHRH Agonists + Immunotherapy (antitelomerase vaccine)	1		

^a^ *mHSPC* metastatic hormone-sensitive prostate cancer

^b^ *mCRPC* metastatic castration resistant prostate cancer

^c^ *TAB* triple androgen blockade (Cyproterone acetate, Bicalutamide, or Flutamide; Leuprolide acetate, Buserelin, Goserelin, or Triptorelin; or Degarelix)

^d^ *CHT* chemotherapy (Docetaxel, Cabazitaxel)

^e^ *ADT* androgen deprivation therapy

^f^ *RT* radiotherapy

Twenty-seven WB-MRI evaluations were performed in patients with metastatic Hormone Sensitive Prostate Cancer (mHSPC) and 23 in mCRPC at the time of examination. The distribution of metastases for each patient at the outset of the study is reported in Table 2. Response to radiotherapy was evaluated in five examinations in mHSPC patients and four examinations amongst the mCRPC patients, with targets of radiotherapy including prostate, spine, pelvic bone, limbs, nodes and other sites.

**Table 2:** Distribution of Metastases by MET-RADS-P Region per Patient at Time of Inclusion

Pat. No.	Bones								Soft Tissues					
	Skull	Cervical spine	Dorsal Spine	Lumbosacral Spine	Pelvis	Thorax	Limbs	Primary Site	Pelvic Nodes	Retroperitoneal Nodes	Other Nodes	Liver	Lungs	Other Sites
1			x	x	x									
2			x	x	x	x	x	x	x	x				
3				x	x			x						
4					x									
5				x	x									
6		x	x	x	x	x				x				x
7		x	x	x	x	x	x	x						
8		x	x	x	x	x	x							
9			x	x	x	x								
10			x	x	x	x	x	x	x	x	x			x
11		x	x		x	x								
12		x	x	x	x	x	x	x						
13			x			x					x			
14					x			x						
15				x	x	x		x						
16		x	x	x	x	x				x	x			
17				x			x	x	x	x	x			
18			x			x				x		x	x	x
19		x	x	x	x	x	x	x						
20			x	x	x	x				x				
21										x	x			
22			x	x	x	x			x	x	x	x	x	
23			x		x		x	x						
24					x									
25					x					x	x			
26				x				x						
27			x	x	x				x	x				
28		x	x	x	x	x	x		x					
29						x				x	x			
30		x	x	x	x		x	x	x	x				
31			x	x			x		x	x	x			

### Radiological findings

The number and distribution of metastatic sites reported was comparable between readers (Table 3), with the Resident Radiologist reporting the presence of metastasis in a total of 251 sites, while the Senior Radiologist reported metastases in 249 sites. Bones were the most frequent sites of metastasis, with both readers reporting bone metastases in 47 out of the 50 WB-MRI evaluations. The second most frequent site of metastasis was lymph nodes, reported in 26 WB-MRI evaluations by Resident Radiologist and in 25 WB-MRI examinations by Senior Radiologist on the basis of enlarged nodes. Both readers reported metastases to liver, lung and pleura, and other sites in a total of 9 WB-MRI.

**Table 3:** Regional Distribution of Metastases by MET-RADS-P Region Reported by the Resident Radiologist (RR) and Senior Radiologist (SR)

	RR	SR	
Bone			
Skull	0	0	
Cervical Spine	16	14	
Dorsal Spine	32	32	
Lumbosacral Spine	33	31	
Pelvis	40	39	
Thorax	28	30	
Limbs	17	17	
Soft Tissues			
Primary Site	18	19	
Pelvic Nodes	11	11	
Retroperitoneal Nodes	24	22	
Other Nodes	14	15	
Liver	6	7	
Lungs	6	6	
Other Sites	6	6	
Total Sites	251	249	

### RAC assessment

The RAC patterns findings for each region are summarized in Supplementary Table E2.

The agreement between readers for primary/dominant RAC pattern (Table 4) was excellent (kappa values between 0.81 and 1.0) for the metastatic findings in cervical, dorsal, lumbosacral spine, pelvis, limbs, lungs and other sites, substantial (kappa values between 0.64 and 0.78) in thorax, retroperitoneal nodes, liver and other nodes, and moderate (kappa = 0.56 (95% confidence interval: 0.14–0.99) for those in pelvic nodes. Inter-observer agreement was not assessed for skull, due to the lack of metastatic findings in our cohort.

**Table 4:** Inter-observer Agreement for Primary/Dominant and Secondary RAC

	Primary RAC Weighted Kappa	Secondary RAC Weighted Kappa
Bone		
Skull	N.E.^a^	N.E.^a^
Cervical Spine	0.86 (0.67–1.00)	0.93 (0.80–1.00)
Dorsal Spine	0.93 (0.86–1.00)	0.79 (0.62–0.96)
Lumbosacral Spine	0.81 (0.69–0.94)	0.44 (0.13–0.74)
Pelvis	0.90 (0.82–0.98)	0.68 (0.47–0.89)
Thorax	0.78 (0.64–0.92)	0.65 (0.39–0.91)
Limbs	0.81 (0.62–0.99)	0.72 (0.38–1.00)
SOFT TISSUES		
Primary Site	0.21 (0.00–0.50)	N.E.^a^
Pelvic Nodes	0.56 (0.14–0.99)	0.66 (0.26–1.00)
Retroperitoneal Nodes	0.64 (0.40–0.89)	0.89 (0.70–1.00)
Other Nodes	0.77 (0.54–1.00)	N.E.^a^
Liver	0.68 (0.28–1.00)	N.E.^a^
Lungs	0.91 (0.73–1.00)	N.E.^a^
Other Sites	1.00 (1.00–1.00)	N.E.^a^

^a^ *N.E*. non evaluable

Agreement was classified by kappa as Excellent (0.81–1.00), Substantial (0.61–0.80), Moderate (0.41–0.60), Fair (0.21–0.40), or None-slight (0.00–0.20)

**Fig. 4:**
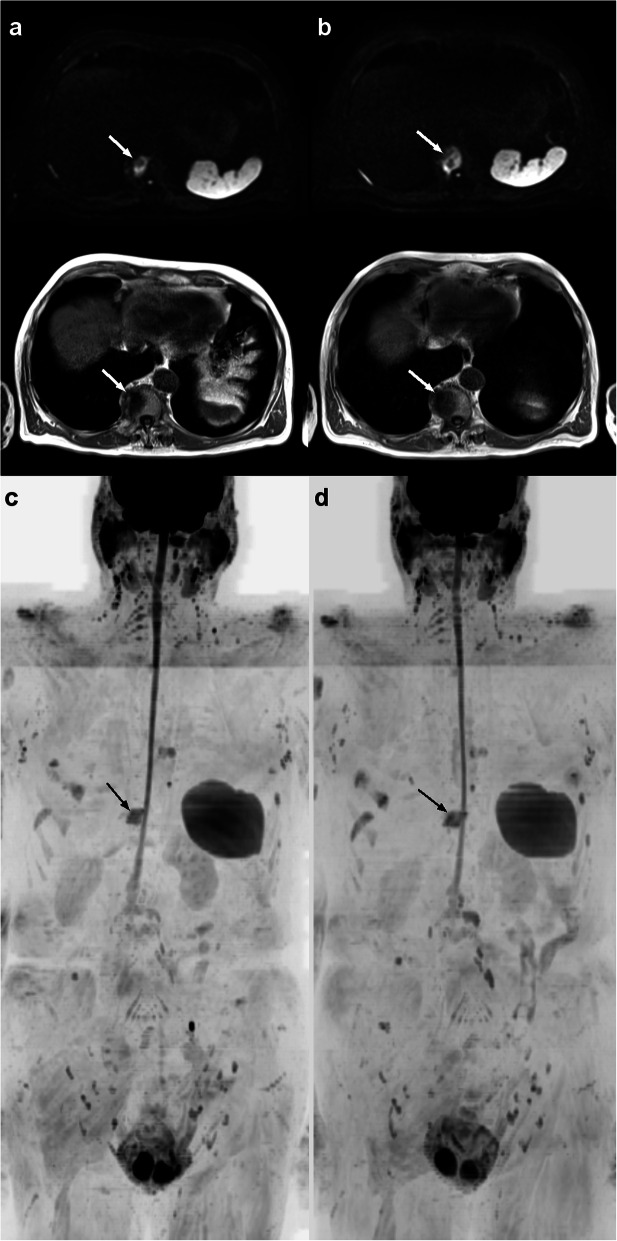
Example images of a 76-year-old man with castration resistant prostate cancer in treatment with bicalutamide and leuprolide acetate where the observers differed in their response assessments of a T11 vertebral body metastasis. The original lesion is seen in **a**) axial DW b900 images (upper) and T1 images (lower) and corresponding **c**) inverted grayscale maximum intensity projections. At a distance of 2 months **b**) and **d**), the Resident Radiologist assigned it as stable (RAC 3) whereas the Senior Radiologist considered it likely to be in progression (RAC 4)

The agreement between readers for secondary RAC pattern (Table 4) was excellent (kappa values of 0.93 (0.80–1.0) and 0.89 (0.70–1.0) respectively) for the metastatic findings in cervical spine and retroperitoneal nodes, substantial (kappa values between 0.65 and 0.79) in dorsal spine, pelvis, thorax, limbs and pelvic nodes, and moderate (kappa = 0.44 (0.13–0.74) for those in lumbosacral spine. A case in which the radiologists differed in their secondary RAC assessment is illustrated in Fig. 4. Inter-observer agreement was not assessed for the remaining regions due to the lack of findings in our cohort.

The primary disease was evaluated only in the 12 patients who had not undergone prostatectomy. The inter-observer agreement in the primary/dominant RAC pattern at the site of primary disease was fair (weighted kappa = 0.21 (0.00–0.50)). Inter-observer agreement for the secondary RAC patterns at the site of primary disease was not evaluated due to the lack of findings in our cohort.

**Fig. 5:**
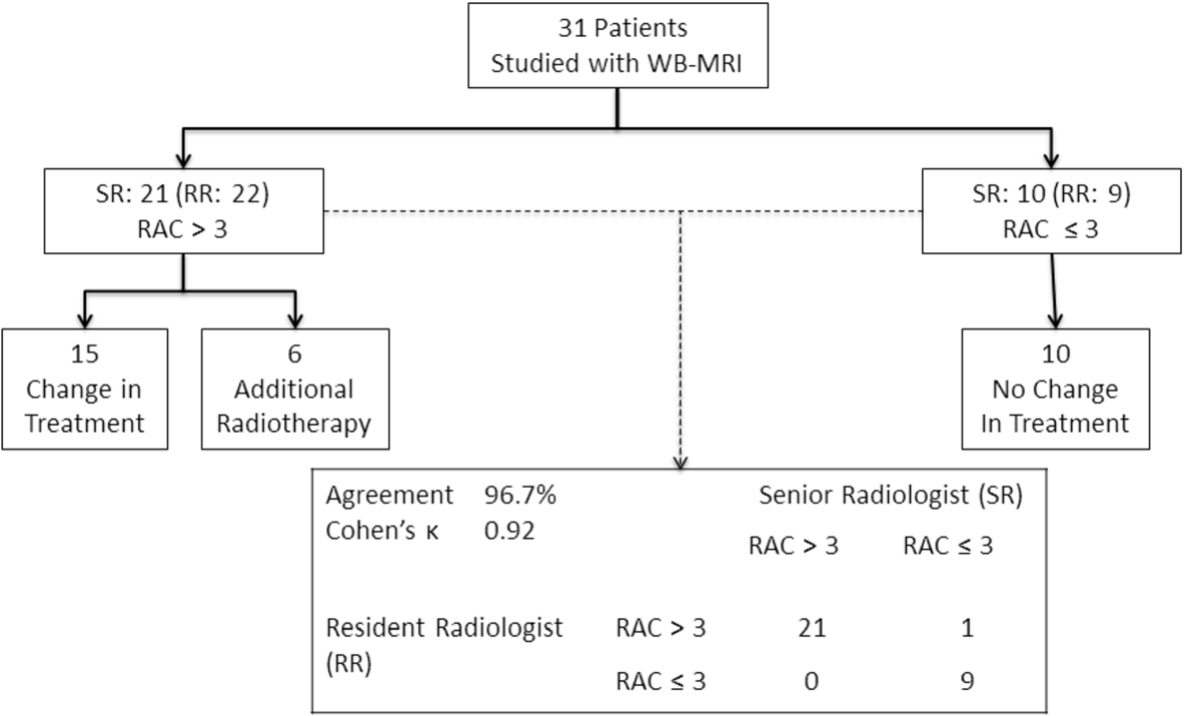
Impact of WB-MRI treatment monitoring on patient management in our 31 patient cohort. Response assessment categories indicating disease that is likely or highly likely to be progressing (RAC > 3) reported by the Senior Radiologist (SR) led to therapy changes for 15 patients, and the addition of radiotherapy in 6 patients. The overall agreement between the Resident Radiologist (RR) and the SR regarding management was 96.7% with a Cohen’s κ of 0.92 (almost perfect agreement) differing only in one case where the Resident Radiologist (RR) assigned a RAC > 3 and the SR had assigned a RAC ≤ 3 (stable disease, or disease likely or highly likely to be responding)

On a per-patient basis (Fig. 5), the radiologists were in agreement for 21 patients regarding the presence of disease likely or highly likely to be progressing (RAC 4 or 5) and for 9 patients regarding the presence of either unchanged disease (RAC 3) or disease likely or highly likely to be responding (RAC 2 or 1). In the remaining patient the Senior Radiologist indicated RAC 1 disease confined to the pelvic nodes whereas the Resident Radiologist indicated the presence of RAC 4 disease. The overall agreement regarding management therefore, was 96.8% with a Cohen’s κ of 0.92 (almost perfect agreement).

## Discussion

Our cohort of 31 patients was relatively balanced between radical prostatectomy and radiotherapy as active treatment of primary tumor (19 vs. 12), and the number of WB-MRI evaluations was balanced between hormone sensitive and castration resistant states (27 vs. 23). Our observations therefore, are unlikely to be biased by treatment modality or clinical state. A majority of our cohort showed bone metastases. This is not surprising as the presence of metastasis was an inclusion criterion of this study, and bone is the most common site of metastasis from prostate cancer.

Overall, the Resident and Senior Radiologists detected a similar number of metastatic sites (251 and 249, respectively). Both detected bone metastases in 47 WB-MRI evaluations, and they were in full agreement for metastases to liver, lung and pleura, and other sites (9 metastases). They agreed well on the number of metastatic nodes (26 by the Resident Radiologist vs. 25 by the Senior Radiologist). Our results suggest, therefore, that the ability to detect of metastases with WB-MRI may not be heavily influenced by reader expertise. This is consistent with the excellent diagnostic performance reported for the detection of metastases with WB-MRI [15] where high sensitivity and specificity of 90 and 95%, respectively has been reported [16, 17].

In our cohort, we found high inter-observer agreement in primary/dominant RAC assessment (excellent or substantial) for all regions except the pelvic nodes. Our inter-observer agreement for response to therapy was higher for bone metastases than for the other body regions. Three considerations likely contribute to this finding. First, our population was composed exclusively of APC patients, for whom the dominant sites of metastases are bone and lymph nodes. Consequently, just four patients had metastases to other soft tissues (liver, lungs, and other sites – Table 2) and thus the inter-observer agreement obtained for these tissues is subject to wide confidence intervals. Second, the literature on response assessment is dominated by studies using RECIST 1.1 criteria [18], which depend primarily on changes in lesion dimensions and are applicable to primary, nodal, and visceral sites, but not for bone metastases. As these same criteria are followed for primary, nodal, and visceral sites under MET-RADS-P, the moderate to substantial inter-observer agreement seen in the primary/dominant RAC assessment of node regions likely reflects known difficulties in assessing nodes with MRI, which is dependent on nodal size assessments [19, 20]. Third, for interpretation of bone metastases, the MET-RADS-P guidelines consider alterations in both size and ADC values, providing extra information that likely increases the performance and inter-observer agreement for bone metastases.

In light of the different levels of readers’ expertise, this suggests that, from a clinical perspective, the MET-RADS-P guidelines constrain the inter-observer variability in the interpretation of WB-MRI in APC. Monitoring the response to treatment of bone disease with CT and BS is difficult because these methods have significant limitations in response assessment [21, 22]. The primary/dominant RAC pattern for bone metastasis, assessed using the MET-RADS-P criteria based on ADC values and morphological features was almost insensitive to the reader’s experience. This is especially important for patients in the castration resistant state, for whom PSA monitoring is a less useful, as up to 30% of patients having clinical or imaging progression without PSA progression [10]. A recent study by Yoshida et al. [23], shows that metastatic burden and tumor characteristics assessed with WB-DWI have the potential to be important prognostic factors in CRPC.

As yet, however, the Prostate Cancer Clinical Trials Working Group 3 does not recommend the routine use of WB-MRI for men with APC treated on clinical trials mainly due to the lack of availability, outcome data, and standardization across global sites [5]. While our findings support the growing evidence that next generation imaging techniques (MRI and PET) may prove to be more accurate for evaluating response of APC to treatment, further validation of the MET-RADS-P guidelines is needed to support change to existing imaging recommendations.

Overall, inter-observer agreement on the secondary RAC pattern was slightly lower than for primary RAC pattern. We attribute this to the fact that secondary RAC pattern assessment involves a minority of the metastases (less than 50% of lesions by definition) in a region, and thus is dependent on the reader’s ability to identify the differences in response present in a small subgroup of metastases (Fig. 4). Assessing the secondary RAC pattern aims to capture discordant (mixed) response following therapy due to clonal adoptions [7]. This differentiation can be clinically important and argues for the need for higher performance in secondary RAC assessment despite a minority of metastases being involved. Missing disease progression at the stage of a secondary RAC finding could translate into delays in shifting to the subsequent treatment, with the patient undergoing ineffective (and expensive) treatment in the interim.

Notably, agreement in the assessment of response at the site of primary tumor was only fair. This assessment is however, dependent on just the 12 patients who had not undergone prostatectomy. This result could be related to the known difficulties in the evaluation of post-treatment imaging of prostate cancer, likely related to the less experience reader suffered more in this context than the experienced.

While MET-RADS-P aims to provide a more complete view of the heterogeneity of response throughout the extent of metastatic disease, as opposed to a single global summary value, ultimately it serves to support decision-making regarding the maintenance or change of therapy. On a per-patient basis (Fig. 5) we found almost perfect agreement (kappa values of 0.92) between the two radiologists. This is interesting in light of the mixed levels of inter-observer agreement seen for various anatomic regions, but likely reflects the good performance of WB-MRI in detecting and monitoring the bone lesions that were frequent in our cohort. Despite the good overall performance regarding disease management, it is worth noting the significant implications for the single patient. In our cohort, there was one case where the Resident Radiologist assigned RAC 4 where the Senior Radiologist indicated RAC 1 disease, that, if supported by clinical evidence could have had an impact on patient management (for example: addition of local radiotherapy or a change in therapy).

An important limitation of the present study is that it involved just two readers is a single institution, with the less experienced Resident Radiologist having been trained by the Senior Radiologist as opposed to the readings being performed by two certified radiologists of different levels of experience in WB-MRI. The Resident Radiologist received training “in-house” in a high volume service, but there is no published data regarding the reader learning curve and requisites for competent reporting in WB-MRI. The large difference in experience likely contributed to the modest inter-observer agreement we observed; particularly for the assessment of secondary RAC where reader training and experience would be expected to play a significant role. The generally good agreement between experienced and inexperienced readers suggests that MET-RADS-P does in fact promote consistent interpretation across readers, but may also be an artifact of the training environment. The fact that the radiologists were not blinded to clinical data or the results of prior examination pairs may have led to bias in their reporting. We believe however, that providing the information in this way reflects clinical practice better than would be achieved with isolated review of examination pairs. Further, as an inclusion criterion was the presence of metastases on a WB-MRI as detected by the Senior Radiologist during clinical routine, the results do not necessarily generalize to the wider population of patients undergoing WB-MRI for metastasis detection and treatment monitoring. Finally, we note that comparator examinations with conventional MRI or CT were not available. Thus, we were not able to verify whether the moderate to substantial agreement between radiologists in regards to lymph nodes is accompanied by inferior sensitivity in detecting lymph node metastases. This is an important consideration for APC patients where lymph node spread is common. Studies with a wider sampling of readers in a larger population of patients, and validation of performance are needed to validate our results.

## Conclusions

We found inter-observer agreement between readers who have different levels of expertise in WB-MRI to be excellent in bone, but mixed in other body regions. Considering the importance of bone metastases assessment in patients with APC, our results favor the use and application of MET-RADS-P in response to the growing clinical need for accurate monitoring of metastasis and improve management in these patients in the era of precision oncology.

## Supplementary information

**Additional file 1 : Table E1.** WB-MRI scanning protocol.

**Additional file 2 : Table E2.** Distribution of primary/dominant and secondary RAC scores by MET-RADS-P region* as reported by the Resident Radiologist (RR) and Senior Radiologist.

## Data Availability

The datasets during and/or analysed during the current study available from the corresponding author on reasonable request.

## Abbreviations

ADC: Apparent diffusion coefficient
ADT: Androgen deprivation therapy
APC: Advanced prostate cancer
AR: Androgen receptor
mCRPC: metastatic castration resistant prostate cancer
MET-RADS-P: METastasis Reporting and Data System for Prostate Cancer
PSA: Prostate-specific antigen
RAC: Response assessment category
WB-MRI: Whole-body magnetic resonance imaging
