# Supplementary material for: Whole-body magnetic resonance imaging (WB-MRI) reporting with the METastasis Reporting and Data System for Prostate Cancer (MET-RADS-P): inter-observer agreement between readers of different expertise levels
Source: Cancer Imaging. 2020 Oct 27;20:77. doi: 10.1186/s40644-020-00350-x (PMC7590732; doi:10.1186/s40644-020-00350-x)
Supplement: Supplementary file 1 — Additional file 1 : Table E1. WB-MRI scanning protocol. [file 40644_2020_350_MOESM1_ESM.docx]

**Table E1. WB-MRI scanning protocol**

| Scanning Parameters (1.5T) | Chest / Abdomen / Pelvis | | | Spine | | POST-PROCESSING* |
| --- | --- | --- | --- | --- | --- | --- |
| Image Contrast | T1-Dixon | T2 | DWI | T1 | T2 STIR | All sequences:   - group separately acquired stacks to form contiguous volumes of each contrast |
| Imaging Sequence | GRE | HASTE | SSH SE EPI | TSE | TSE |  |
| Orientation | Axial | Axial | Axial | Sagittal | Sagittal |  |
| Echo / Repetition Time (ms) | 2.39,4.77 / 6.65 | 74 / 800 | 62 / 6550 | 9.3 / 350 | 60 / 2560 |  |
| Field of view (mm) | 430 | 430 | 430 | 400 | 400 | DWI:   - Calculate ADC - Generate rotating MIPs from high b-value images, display using inverted grey-scale |
| Matrix | 352 x 209 | 320 x 175 | 132 x 120 | 448 x 224 | 320 x 160 |  |
| Slices per Station / Stations | 72 / 4 | 191 / 1 | 50 / 4 | 16 / 2 | 16 / 2 |  |
| Flip angle (°) | 20.5 | 90 | 90 | 90 | 90 |  |
| Slice Thickness / Gap (mm) | 3.5 / 0 | 5 /1 | 5 / 0 | 4 / 0.4 | 4 / 0.4 |  |
| Fat Suppression | - | - | STIR | - | STIR |  |
| Respiratory Control | Breath-Hold | Breath-Hold |  |  | - | DIXON:   - Calculate in, out, water and fat |
| b-values (s/mm^2^) | - | - | 50, 900 | - | - |  |
| Acquisition Time (min:sec) | 1:04 | 2:46 | 15:04 | 3:32 | 3:10 |  |

GRE – Gradient-echo; HASTE – Half-Fourier Acquisition Single-shot Turbo-spin Echo; TSE – Turbo-Spin Echo; SSH SE EPI – Single-Shot Spin-Echo EchoPlanar Imaging; STIR – Short Tau Inversion Recovery.

*post processing according to MET-RADS-P guidelines (9)
