# Supplementary material for: Whole-body magnetic resonance imaging (WB-MRI) reporting with the METastasis Reporting and Data System for Prostate Cancer (MET-RADS-P): inter-observer agreement between readers of different expertise levels
Source: Cancer Imaging. 2020 Oct 27;20:77. doi: 10.1186/s40644-020-00350-x (PMC7590732; doi:10.1186/s40644-020-00350-x)
Supplement: Supplementary file 2 — Additional file 2 : Table E2. Distribution of primary/dominant and secondary RAC scores by MET-RADS-P region* as reported by the Resident Radiologist (RR) and Senior Radiologist. [file 40644_2020_350_MOESM2_ESM.docx]

**Table E2. Distribution of primary/dominant and secondary RAC scores by MET-RADS-P**

**region* as reported by the Resident Radiologist (RR) and Senior Radiologist (SR)**

| Bone Region | Sites Reported | |  | Soft Tissue Region | Sites Reported | |
| --- | --- | --- | --- | --- | --- | --- |
|  | RR | SR |  |  | RR | SR |
| SKULL | **0** | **0** |  | Primary Site | **18** | **20** |
| Cervical Spine | **28** | **25** |  | *RAC 1* | *0* | *2* |
| *RAC 1* | *10* | *7* |  | *RAC 2* | *5* | *4* |
| *RAC 2* | *4* | *3* |  | *RAC 3* | *13* | *11* |
| *RAC 3* | *8* | *10* |  | *RAC 4* | *0* | *1* |
| *RAC 4* | *3* | *2* |  | *RAC 5* | *0* | *2* |
| *RAC 5* | *3* | *3* |  | Pelvic Nodes | **19** | **19** |
| Dorsal Spine | **57** | **56** |  | *RAC 1* | *5* | *7* |
| *RAC 1* | *13* | *14* |  | *RAC 2* | *4* | *3* |
| *RAC 2* | *15* | *14* |  | *RAC 3* | *2* | *3* |
| *RAC 3* | *11* | *11* |  | *RAC 4* | *4* | *2* |
| *RAC 4* | *4* | *3* |  | *RAC 5* | *4* | *4* |
| *RAC 5* | *14* | *14* |  | Retroperitoneal Nodes | **32** | **29** |
| Lumbosacral Spine | **56** | **52** |  | *RAC 1* | *3* | *5* |
| *RAC 1* | *9* | *13* |  | *RAC 2* | *10* | *9* |
| *RAC 2* | *13* | *11* |  | *RAC 3* | *9* | *5* |
| *RAC 3* | *11* | *7* |  | *RAC 4* | *5* | *2* |
| *RAC 4* | *5* | *5* |  | *RAC 5* | *5* | *8* |
| *RAC 5* | *18* | *16* |  | Other Nodes | **19** | **19** |
| Pelvis | **75** | **73** |  | *RAC 1* | *2* | *3* |
| *RAC 1* | *25* | *28* |  | *RAC 2* | *7* | *9* |
| *RAC 2* | *12* | *13* |  | *RAC 3* | *7* | *3* |
| *RAC 3* | *12* | *9* |  | *RAC 4* | *1* | *2* |
| *RAC 4* | *8* | *7* |  | *RAC 5* | *2* | *2* |
| *RAC 5* | *18* | *16* |  | Liver | **7** | **8** |
| Thorax | **49** | **52** |  | *RAC 1* | *0* | *1* |
| *RAC 1* | *13* | *15* |  | *RAC 2* | *3* | *2* |
| *RAC 2* | *6* | *6* |  | *RAC 3* | *1* | *1* |
| *RAC 3* | *14* | *12* |  | *RAC 4* | *0* | *0* |
| *RAC 4* | *5* | *7* |  | *RAC 5* | *3* | *4* |
| *RAC 5* | *11* | *12* |  | Lungs | **7** | **6** |
| Limbs | **24** | **26** |  | *RAC 1* | *3* | *4* |
| *RAC 1* | *3* | *3* |  | *RAC 2* | *1* | *0* |
| *RAC 2* | *2* | *5* |  | *RAC 3* | *0* | *0* |
| *RAC 3* | *8* | *11* |  | *RAC 4* | *0* | *0* |
| *RAC 4* | *4* | *2* |  | *RAC 5* | *3* | *2* |
| *RAC 5* | *7* | *5* |  | Other sites | **8** | **7** |
|  |  |  |  | *RAC 1* | *0* | *0* |
|  |  |  |  | *RAC 2* | *3* | *3* |
|  |  |  |  | *RAC 3* | *0* | *0* |
|  |  |  |  | *RAC 4* | *0* | *0* |
|  |  |  |  | *RAC 5* | *5* | *4* |

* for each region the total number of sites of metastases is given (in bold), followed by their distribution across RAC scores (in italics).
